# Optimized Design, Monitoring System Development and Experiment for a Long-Belt Finger-Clip Precision Corn Seed Metering Device

**DOI:** 10.3389/fpls.2022.814747

**Published:** 2022-01-28

**Authors:** Han Tang, Changsu Xu, Ziming Wang, Qi Wang, Jinwu Wang

**Affiliations:** College of Engineering, Northeast Agricultural University, Harbin, China

**Keywords:** seed metering device, optimization design, monitoring system, performance of precision, experiment

## Abstract

To solve multiple problems, such as the poor seeding process stability in the conventional finger-clip precision corn seed metering device and the inability to monitor the seeding effect, a long-belt finger-clip precision seed metering device was optimized and designed. The overall structure and working principle were described, and the mechanism of smooth transport and delivery was analyzed. A diffuse reflection photoelectric sensor and rectangular optical fiber sensor were used to monitor the number of corn seeds in the seeding process, and the states of multiple and miss seeding were calculated. A corn seeding quality monitoring system was designed. In this study, the seed metering performance of the long-belt finger-clip precision seed metering device was compared to that of the conventional finger-clip precision corn seed metering device. It was shown that the reseeding index, the miss-seeding index and the coefficient of variation can be effectively reduced with increasing seed metering tray speed. At the maximum speed of 65r/min, the qualified index increased from 75.75 to 84.70%, the reseeding index decreased from 13.66 to 8.49%, the miss-seeding index decreased from 10.59 to 6.81%, and the coefficient of variation decreased from 20.69 to 6.83%. The variations of these four evaluation parameters with the seed metering tray rotating speed were analyzed. Furthermore, the effects of the seeding frequency and seeding speed on the four evaluation parameters were studied through single factor and variance analyses. The results showed that the relative errors of the qualified index, the reseeding index, the miss-seeding index and the seeding amount increased gradually with the increase in the seed metering tray rotating speed, and the monitoring accuracy of the sensor decreased gradually. The accuracy of sensor monitoring decreased with increasing seeding frequency and seeding speed. This study provides an optimized scheme for the smooth delivery and movement of conventional seed metering devices and provides a technical reference for the development and design of monitoring systems with multiple index and the miss-seeding index of seed metering devices.

## Introduction

Precision seeding has the advantages of conserving seeds, reducing labor intensity and improving operation efficiency, comprising one of the important trends for agricultural development in the future (Yazgi and Degirmencioglu, 2014; Xing et al., 2020). As the core working component, precision seed metering devices are mainly divided into mechanical and pneumatic types (Navid et al., 2011; Miller et al., 2012). The pneumatic seed metering device has the characteristics of good universality and high working speed, but it needs additional air source, has complex structure and high failure rate. In addition, seeds need to be strictly graded. Compared with pneumatic seed metering device, mechanical seed metering device is widely used because of its simple structure, convenient maintenance, low cost, no pressure air source and no sealing. At present, the common mechanical seed metering devices mainly include socket-wheel type, disk type, spoon type and finger-clip type. In the process of corn sowing, finger-clip seed metering device has the characteristics of low damage rate and good adaptability. It is the most widely used among the types of mechanical seed metering device (Hossen et al., 2014; Haque et al., 2016; Soyoye, 2020). The research on finger clip precision corn seed metering device is of great significance to popularize precision sowing technology. However, the problem of poor stability in the transportation and delivery process, caused by the violent vibration of working components, is a common problem in mechanical precision seed metering devices (Zhao et al., 2020). At the same time, the seed metering device process is closed, so it is impossible to directly observe the effect of seeding. If such problems in seed metering devices are not found in time during a large-scale seeder operation process, periodic large-area multiple seeding and miss seeding will occur because of the fast working speed and the wide seeding range (Wang et al., 2020). Therefore, it is necessary to monitor the working quality of the seed metering device to ensure reliability and stability in the process of precision seeding.

The monitoring of the seeding quality is the basis for not only the automation and intelligence of the seeding link but also the reliable guarantee of signal feedback and equipment research along with the development of the follow-up seeding link. A large number of scholars have devoted themselves to seeding quality monitoring research, which includes high-speed photography, piezoelectric sensing, and photoelectric monitoring (Zhao and Jian, 2005; Chen et al., 2009; Li et al., 2010). Among these methods, the high-speed photography method has the advantage of high monitoring accuracy. Karayel et al. (2006) used high-speed camera and image processing technologies to obtain the seed distance and seed distribution uniformity, then judged the qualification of the seed metering device based on the seed distance and uniformity. However, this method involves a large amount of image data and expensive equipment, so it is difficult to popularize and apply in the actual working process. The piezoelectric sensing method identifies the seeding amount by monitoring the change in pulse voltage produced by the seed hitting a piezoelectric film or piezoelectric ceramic (Zhang et al., 2011). Wang et al. (2019) developed an impact seeding amount monitoring sensor for rice hill direct seeding; this sensor can effectively identify the seeding amount of rice seeds hitting the piezoelectric film. Ding et al. (2017) developed a small seed size seeding amount sensor based on the piezoelectric impact principle; this sensor realized the real-time monitoring of the seed metering frequency and total seed metering amount. Huang et al. (2013) developed a precision corn seeding monitoring system based on a polyvinylidene fluoride piezoelectric film, and the monitoring accuracy of miss-seeding rate was 96%. The technical difficulty of the piezoelectric sensing method is related to the selection of appropriate sensitive materials according to the physical characteristics of different seeds, where the installation positions of sensitive materials have higher requirements. Compared with the high-speed photography method and the piezoelectric sensor method, the photoelectric monitoring method has the advantages of a simple sensor structure, fast response speed, and easy development and configuration; therefore, this method has gradually become the focus of research (Al-Mallahi and Kataoka, 2016; Besharati et al., 2019). Karimi et al. (2017, 2019) built a seeding monitoring device and a seeding monitoring system with infrared laser diode array sensors and realized the measurement of seed flow. However, the array can monitor only the seed information in a single direction, easily producing monitoring errors. Kumar and Raheman (2018) arranged an infrared light-emitting diode (LED) as a ring. Such infrared light covered the whole section of the seeding tube, effectively improving the monitoring accuracy and overall accuracy. Due to the different delivery and transport modes of different seed metering devices, a diversity in the seed falling posture often appears in the seeding process (Liu et al., 2018). One of the most common situations is the overlap in the process of seed falling, which will lead to the sensor unable to effectively monitor the number of seeds and the problem of low monitoring accuracy.

Limited by the characteristics and performance of the sensor, the use of a single sensor can meet only the seeding conditions of small flow and low speed. With the increase in the speed of the seed metering tray, a single sensor cannot achieve accurate and effective monitoring. Zhang et al. (2013) installed LEDs and photosensitive resistors in the seed guide tube of a soybean seeder to detect the seeding condition based on the blocked light when the seeds fell. Lan et al. (1999) used an LED with a diameter of 3 mm and a phototransistor photosensor to measure the spacing of small seeds from the seed metering device. Qiu et al. (2019) designed an operation quality monitoring system of a small particle size seeder based on a photoelectric sensor for rectangular infrared detection and a complementary metal-oxide semiconductor (CMOS) image sensor. Visualization of this seeding operation monitoring was realized by seed image acquisition and a photoelectric sensor. Technology based on multisensor information fusion can greatly improve the effective monitoring of seeding quality under multiple working conditions, but the installation positions, configuration forms and monitoring objects of multisensors are not universal. The problem of multisensor configuration and development for specific crop monitoring needs to be solved.

To solve problems such as the poor seeding process stability in the conventional finger-clip precision seed metering device used for corn and the inability to monitor the seeding effect, the long-belt finger-clip precision seed metering device used for corn was optimized and designed. The mechanism of smooth transport and delivery was analyzed. A diffuse reflection photoelectric sensor and a rectangular optical fiber sensor were used to monitor the seeding state information. Based on these sensors, a quality monitoring system for precision corn seed metering devices was developed. The effectiveness of the long-belt finger-clip precision corn seed metering device for smooth migration and the reliability of the monitoring system were verified by comparative experiments, single factor experiments and variance analysis. This study provides an optimized scheme for smooth delivery and movement in conventional seed metering devices and provides a technical reference for monitoring system development and design with multiple index and the miss-seeding index of seed metering devices.

## Materials and Methods

### Overall Structure and Working Principle of the Long-Belt Finger-Clip Precision Corn Seed Metering Device and Monitoring System

**FIGURE 1:**
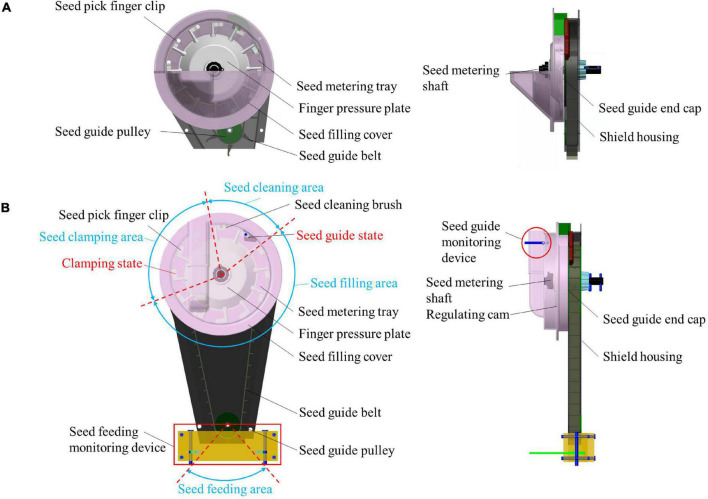
Overall structure. **(A)** Overall structure of conventional finger-clip precision corn seed metering device. **(B)** Overall structure of long-belt finger-clip precision corn seed metering device.

Conventional finger-clip precision corn seed metering device is mainly composed of a seed pick finger clip, seed guide pulley, seed metering tray, finger pressure plate, seed filling cover, seed guide belt, seed metering shaft, seed guide end cap, shield housing. The overall structure is shown in Figure 1A. The conventional finger-clip precision corn seed metering device is equipped with a seed guide tube under it, so it is unable to sowing at zero speed, resulting in the reduction of seed metering performance when the seeds bounce and collide. In addition, the structural configuration cannot meet the layout requirements of sensors. In this study, the seed guide tube structure was replaced by the lengthened seed guide belt, which simplified the overall structure. At the same time, the research was carried out from the aspect of stable delivery mechanism to achieve the effect of zero speed seed delivery. A long-belt finger-clip precision corn seed metering device is designed. It is mainly composed of a seed guide pulley, seed guide belt, seed filling cover, finger pressure plate, seed metering tray, seed cleaning brush, seed-pick finger-clip, seed feeding monitoring device, shield housing, seed guide end cap, seed guide monitoring device, regulating cam and seed metering shaft. The overall structure is shown in Figure 1B. The parameters of long-belt finger-clip precision corn seed metering device is shown in Table 1. Among these components, the finger pressure plate and the regulating cam are two of the key working components in the seed metering device, and the rationality of the structure configuration directly affects the working quality of the seed metering device. The finger pressure plate is a combination of 12 finger clips connected by fine-tuning springs (the number of finger clips can be adjusted freely, and up to 12). The finger pressure plate and regulating cam are installed on the inside of the finger clamping plate in turn. The seed metering tray is made of galvanized steel to increase the friction characteristics of the corn seeds. The seed guide belt is made of rubber, and its circumference is equipped with 12 inclined blades. The 12 seed guide rooms are formed with the seed guide end cap and the machine shell. The seed cleaning brush is made of bristles, and the angle of the brush can be artificially adjusted to control the seed cleaning degree. The seed guide monitoring device is situated at the seed guide port, and a diffuse reflection photoelectric sensor is used to monitor the number of theoretical seeds (the number of seed-pick finger-clips is monitored through the diffuse reflection photoelectric sensor, were a seed-pick finger-clip carries a corn seed in theory). The seed feeding monitoring device is arranged under the seed feeding port, and a rectangular optical fiber sensor is used to monitor the number of seed guide belts delivered to the soil.

**TABLE 1:** Parameters of long-belt finger-clip precision corn seed metering device.

Item	Value	Unit
Number of finger clips	0−12	/
Diameter of seed guide pulley	50	mm
Diameter of seed guide pulley I	200	mm
Seed guide belt spacing	40	mm
Seed metering disk speed	0−65	r/min

The working area of the seed metering device can be divided into four stages: the seed filling area, the seed holding area, the seed cleaning area and the seed feeding area. During operation, the seed filling room is filled with the corn seeds from the seed box. The walking wheel of the machine transmits the power to the seed metering shaft through chain transmission and drives the finger pressure plate and the seed-pick finger-clip to rotate. The fixed cam and the fine-tuning spring work together to control the timing of the finger clip opening and closing. When the seed-pick finger-clip is opened, the seed is filled and clamped in the seed filling area. When the seed-pick finger-clip is closed, multiple seeds are withheld from the seed filling area to complete the seed filling process. The seed-pick finger-clip rotates and moves smoothly in the seed holding area. When the seed is moved to the seed cleaning area, the seed cleaning brush removes the multiple seeds and completes the seed cleaning process. A single grain of corn is placed in the seed guide room on the back of the seed metering device from the seed guide mouth, and the seed guide belt rotates synchronously with the finger pressure plate. A single seed is smoothly deposited into the soil to complete the process of seeding, and precision seeding is achieved. To realize the quality monitoring of a precision seed metering device, a diffuse reflection photoelectric sensor and a rectangular optical fiber sensor are set up under the seed guide port and the seed feeding port, respectively. When the seed is transported and delivered, the sensor is shielded to return a low-level signal. According to the pulse signal returns by the sensor and processes by the single-chip microcomputer, the seed falling time interval is counted and compared to the set theoretical time interval. The reseeding index and miss-seeding index in the process of precision seeding are calculated.

### Analysis of the Mechanism for Stable Transport and Delivery

**FIGURE 2:**
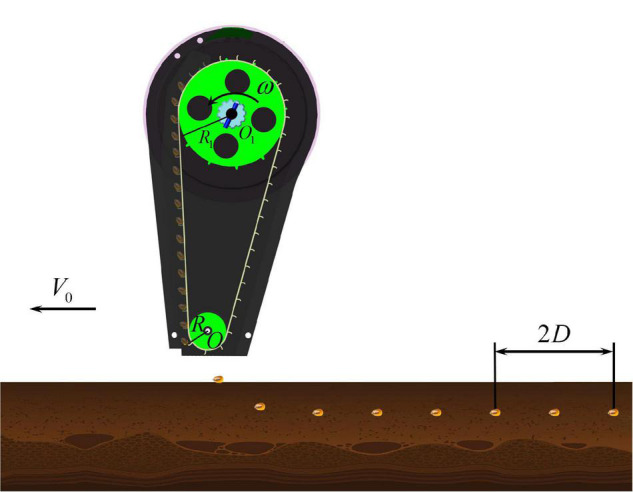
Schematic diagram of smooth transport and delivery. *D* is the corn seed spacing, mm; *V*_0_ is the working speed of the machines, mm/s; ω is the angular velocity of the seed metering shaft, rad/s.

The lengthened seed guide belt of the long-belt finger-clip precision corn seed metering device can reduce the delivery height of the seed to the soil and slow down the bouncing collision frequency between the seed and the soil in the process of high-speed precision seeding. The seeding accuracy, uniformity and horizontal and vertical straightness are improved. In the seeding process, the seed metering shaft drives the seed metering tray to rotate, and the seed-pick finger-clip rotates the single seed counterclockwise; the single seed is then smoothly transported to the seed guide mouth for seeding. The first migration operation is realized. The seed is thrown onto the seed guide belt, which rotates synchronously with the seed metering tray through the seed guide mouth and moves smoothly to the seed delivery point under the action of gravity and the supporting force. The zero speed in low position of the seed to the soil is realized. The second migration operation is realized. The relative speed of the seeds falling into the seed ditch is offset by secondary delivery, and smooth seed transport and delivery is realized, as shown in Figure 2.

**FIGURE 3:**
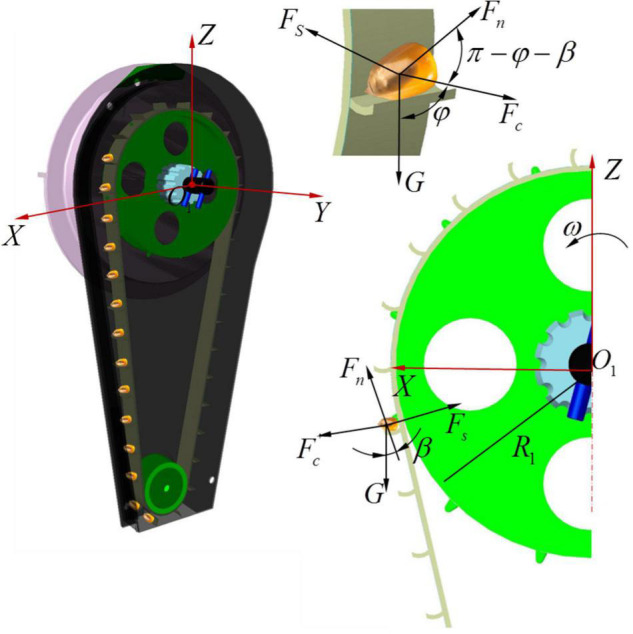
Force analysis diagram of the stable transport and delivery process.

To study the stationarity of the seeds in the transport and delivery stage, the motion state of the seeds is mechanically analyzed and the critical conditions of the relative balance between the seeds and the seed guide blade are studied (Wang et al., 2017). Taking the rotating center of the seed metering shaft as the coordinate origin *O*_1_, the spatial Cartesian coordinate system *XYZ* is established, as shown in Figure 3. When a single seed enters the seed guide room through the seed guide port, the force of the seed is analyzed when it rotates counterclockwise with the seed guide belt. The seeds are affected by the spatial force system composed of the centrifugal force *F*_*c*_, the supporting force of the blade *F*_*n*_, the friction force of the blade *F*_*s*_ and the seed gravity *G*.

If the relative balance between the seed and the seed guide blade is ensured and the seed is not thrown away from the seed guide blade, the force along the slip direction of the seed relative to the blade should be satisfied in the rotating plane *XO*_1_*Z* of the seed guide belt.


(1) $$F_{c}+G\,\cos\phi +F_{n}\,\cos\left(\pi -\beta -\phi \right)\leq F_{s}\,\cos\beta$$

Among these values,


(2) {Fc=m⁢ω2⁢R1Fn=m⁢g⁢ sin⁡βFs=μ⁢Fn

where *m* is the mass of the corn seed, mg; ϕ is the relative rotation angle of the seed guide blade, (°); β is the structural inclination angle of the seed guide blade, (°); μ is the friction coefficient between the corn seed and the seed guide blade, set to 0.15; *R*_*1*_ is the radius of the seed guide pulley I, mm; and *F*_*c*_ is the centrifugal force of the corn seeds, N.

Equation (3) is obtained by the combination of Equation (1) and Equation (2).


(3) m⁢ω2⁢R1+m⁢g⁢ cos⁡ϕ≤m⁢g⁢ sin⁡β⁢(μ⁢cos⁡β+cos⁡(β+ϕ))

To solve the limiting value of the seed metering speed under the critical slip state, Equation (3) is arranged as follows:


(4) ω≤g⁢ sin⁡β⁢(μ⁢cos⁡β+cos⁡(β+ϕ))-g⁢ cos⁡ϕR1

When the speed of the seed metering tray satisfies Equation (4), the corn seed and the seed guide belt remain relatively static and relative slip does not occur. Equation (4) shows that the factors affecting the speed limit of the smooth seed movement and delivery are related to the relative rotation angle of the seed guide blade, the structural inclination angle of the seed guide blade and the radius of the seed guide pulley I. Moreover, this limit is related to the friction coefficient between the corn seeds and the seed guide blade but has nothing to do with the mass of the corn seeds.

### Design of the Seeding Monitoring System

The hardware system and software system of the seeding monitoring system were designed to accurately monitor the important seed metering indexes, such as the qualified index, reseeding index and miss-seeding index, in the metering process of a long-belt finger-clip precision corn seed metering device.

#### Hardware System Design

**FIGURE 4:**
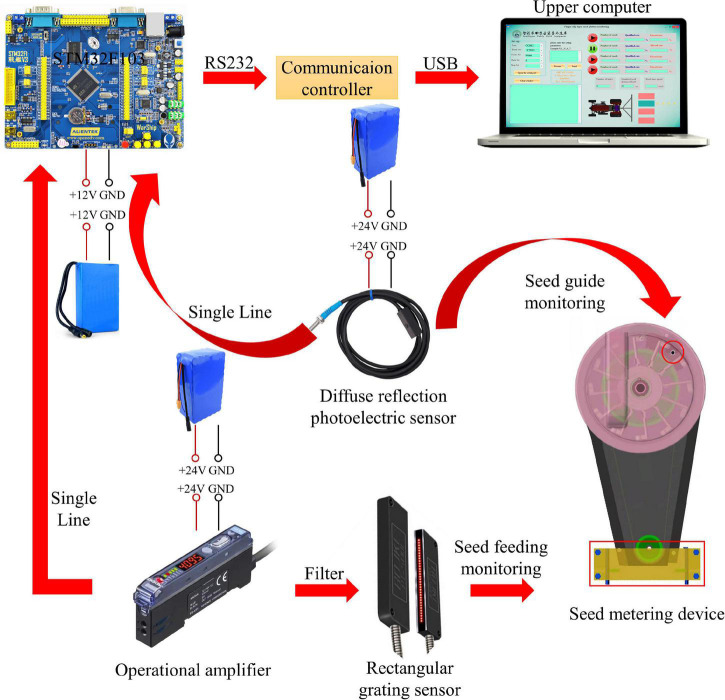
Hardware system.

The hardware system is mainly composed of a STM32F103 single-chip microcomputer, universal serial bus (USB) to transistor-transistor logic (TTL) chip, upper computer, integrated operational amplifier, diffuse reflection photoelectric sensor, rectangular grating sensor and fixed device, as shown in Figure 4. Each part is electrically connected through the communication lines, the signal lines and the power lines to complete the information exchange. Among these components, the power lines are used for the power supply and connection between each piece of equipment, where the power supply is the direct current (DC) 12 V vehicle battery. The communication line adopts the RS232 serial communication network, as designed according to the communication protocol, which was beneficial to the expansion of the system. A rectangular grating sensor adopts the principle of infrared radiation, which changes the incident light path in the seeding state and changes the light intensity received by the infrared receiver. The signal is processed into a pulse signal by the signal amplifier. The single-chip timer (time 4, time 5) is configured to input capture mode, and the corresponding input/output (IO) port is set as the input mode. Thus, the pulse signal was obtained, and the planting parameters were calculated. At the same time, this system cooperated with the diffuse reflection photoelectric sensor installed on the seed guide port, the times of the seed-pick finger-clip passing through the seed guide port were monitored and the rotating speed was calculated according to the reflection principle of light. Based on the times and rotating speed of the seed-pick finger-clip monitored by the diffuse reflection photoelectric sensor, multiple seeding occurred when the data monitored by the rectangular grating sensor were larger than those monitored by the diffuse reflection photoelectric sensor. When the data monitored by the rectangular grating sensor were smaller than those monitored by the diffuse reflection photoelectric sensor, miss seeding occurred. The data detected by the sensor were transmitted to the STM32F103 single-chip microcomputer through the signal line for control and calculation, and the serial port signal was collected by the upper computer through the USB to TTL chip to obtain the seeding information.

#### Software System Design

Seed metering monitoring was mainly realized by monitoring the number of input seeds, the number of rotations and the rotating speed of the seed-pick finger-clip through the seed guide port. Then, a diffuse reflection photoelectric sensor and a rectangular grating sensor converted this information into a pulse signal, and the system counted the number of seeds and the number of times the seed-pick finger-clip (theoretical planting number) was placed in the seed guide port by monitoring the falling edge of the pulse signal. Timing was determined by monitoring the rising edge and falling edge of a pair of pulse signals. The seeding monitoring sensor time was recorded as *T*_*A*_. The interval time of the seed guide monitoring sensor was recorded as *T*_*B*_.

**FIGURE 5:**
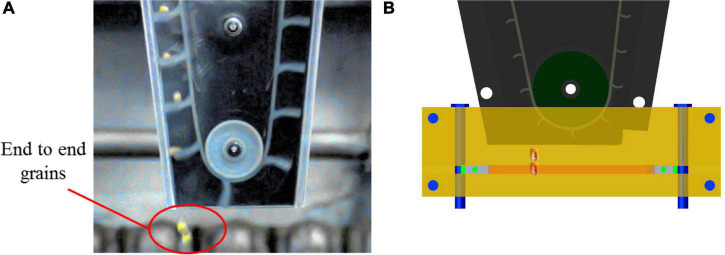
Schematic diagram of multiple seeding. **(A)** Rseeding in high-speed camera observation. **(B)** Principle of reseeding monitoring.

**FIGURE 6:**
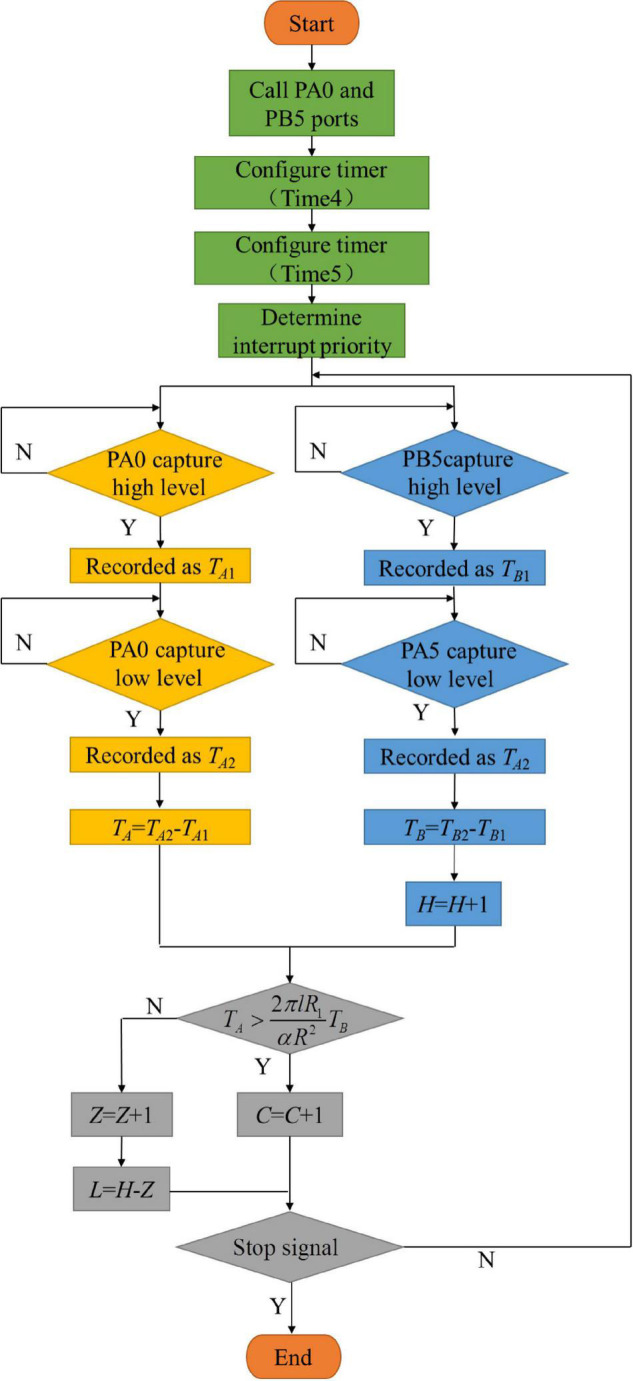
Program flow chart. *L* is the miss-seeding amount; *C* is the reseeding amount; *H* is the theoretical seeding amount; and *Z* is the total amount of seeding.

According to the high-speed camera experiment, the rectangular grating sensor received two pairs of pulse signals in succession when multiple seeding events occurred. However, the diffuse reflection photoelectric sensor received a pair of pulse signals only once, and the number of multiple seedings was increased by one. The end-to-end connection occurred in the vertical plane during the fall of the multiple seeding, as shown in Figure 5. The interval time of the rectangular grating sensor *T*_*A*_ increased, which resulted in the omission of multiple seeding counts. Therefore, the number of multiple seedings was increased by one when Equation (5) was satisfied. The system program flow chart is shown in Figure 6. Firstly, enable the logic pins of single chip PA0 and PB5, and the corresponding timers (Time4 and Time5) are configured to enable the single chip microcomputer to receive external signals and have timing ability. In order to determine the sequence of receiving or transmitting signals, the priority of receiving interrupt and transmitting interrupt is set. At this time, PA0 and PB5 wait for the external signal to trigger. When any pin voltage becomes high, the timer register stores the current time *T*_*A*1_ or *T*_*B*1_. When the external signal disappears, the pin voltage becomes low, and the timer register stores the current time *T*_*A*2_ or *T*_*B*2_. The CPU calculates the signal receiving time *T*_*A*_ or *T*_*B*_, and the theoretical sowing amount is increased by one grain. The CPU determines whether there is a multiple seeding amount and miss seeding amount according to the discrimination relationship. Finally, the CPU determines whether the stop signal is received. If the stop signal is received, the program stops, otherwise it enters the next cycle.


(5) $$T_{A}>\frac{2\,\pi \,l\,R_{1}}{\alpha \,R^{2}}\,T_{B}$$

where *T*_*A*_ is the total time of the seed occlusion rectangular grating sensor, s; *T*_*B*_ is the total time based on the finger clip occlusion diffuse reflection photoelectric sensor, s; *l* is the length of the seed, mm, the vernier caliper was used to measure the length of 1,000 “Demeiya No. 1” maize seeds widely planted in Northeast China, and the average value was taken, taking 9.35mm; and α is the rotation angle of the seed-pick finger-clip, (°).

**FIGURE 7:**
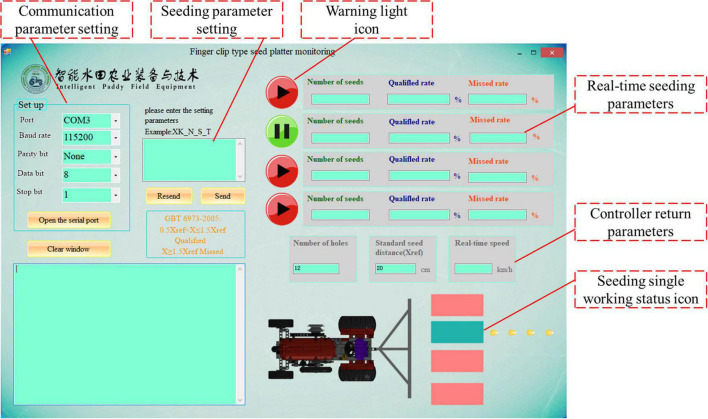
Software interface.

In this study, a Windows-form application program was developed and designed based on the C# language; this program can display the monitoring seeding parameters in real time. Additionally, when the phenomena of multiple seeding and miss seeding occurred, a seeding fault alarm occurred in the software interface. The software interface is shown in Figure 7.

According to the seeding function, the software interface can be divided into four areas: the communication string number setting, seed parameter setting, operation parameter monitoring and miss seeding alarm. Among these areas, the communication string number setting area is used for the selection and opening and closing of the working string number for the equipment communication. The parameter setting area is used to design the relevant parameters, such as the length of the finger clip. After the parameter design is completed, it is saved to the running memory, and the corresponding parameters are read when the program is running. The monitoring area of the operation parameters includes the number of seedlings, the theoretical number of seedlings, the interval seeding time and the rotation speed of the seed metering tray. Among these parameters, the number of seedlings is used to display the seeding number of each row for the seed metering device. The theoretical number of seedlings is used to determine the number of seedlings in each row under normal operating conditions (without miss seeding and multiple seedlings). The phenomenon of multiple seeding is determined through the rotational speed of the seed metering tray and the seeding time interval. When miss seeding occurs continuously, the miss seeding alarm displays a flashing red light to inform the user. Through the abovementioned method, the online monitoring of the qualified index, the reseeding index and the miss index can be realized.

### Experiment

To compare the seeding performances of the long-belt finger-clip precision corn seed metering device and conventional finger-clip precision corn seed metering device and to detect the accuracy of the designed monitoring system, a bench experiment was carried out on seeding performance. The experimental site was the Seeding Performance Laboratory of Northeast Agricultural University. Demeya No. 1 corn seed was selected as the experimental variety. The corresponding 1,000-grain mass was 281.12 g, the average density was 1.154 g/cm^3^, and the average length was 9.27 mm, width was 7.40 mm, and thickness was 4.11 mm (the average values were measured based on 100 seeds). The experimental device was the JPS-12 seed metering device performance experiment bench developed by the Heilongjiang Agricultural Machinery Engineering Research Institute. According to GB/T6973-2005 “Experiment method of single seed (Precision) seeder,” the experiment factors were selected as the rotating speed of seed metering tray, which were 15 r/min, 25 r/min, 35 r/min, 45 r/min, 55 r/min, and 65 r/min, respectively. The experimental indexes included the qualified index, the reseeding index, the miss-seeding index and the coefficient of variation to evaluate the seeding stability of the seed metering device. Additionally, the accuracy of the monitoring sensor was evaluated based on the relative deviation of the qualified index, the reseeding index, the miss-seeding index and seeding amount. The greater the relative deviation, the lower the monitoring accuracy of the seed metering device. There were 1,000 seeds measured in each group, the experiment was repeated three times, and the results were averaged. During the experiment, the spacing between adjacent corn seeds was measured, and the number of seeds was counted to calculate each index. The calculation equation of each index was as follows:


(6) $$\left\{ \begin{array}{c} Q=\frac{n_{0}}{N}\times 100\%\\ M=\frac{n_{1}}{N}\times 100\%\\ E=\frac{n_{2}}{N}\times 100\%\\ V=\sqrt{\frac{\sum \left(x-\bar{x}\right)}{\left(n^{\prime }-1\right)\,{\bar{x}}^{2}}}\times 100\%\\ Q^{\prime }=\frac{\left|n_{0}^{\prime }-n_{0}\right|}{n_{0}}\times 100\%\\ M^{\prime }=\frac{\left|n_{1}^{\prime }-n_{1}\right|}{n_{1}}\times 100\%\\ E^{\prime }=\frac{\left|n_{2}^{\prime }-n_{2}\right|}{n_{2}}\times 100\%\\ S=\frac{n_{a}-n_{b}}{n_{a}}\times 100\% \end{array}\right.$$

where *Q* is the qualified index, %; *M* is the reseeding index, %; *E* is the miss-seeding index, %; *V* is the coefficient of variation, %; *Q*′ is the relative error of the qualified index, %; *M*′ is the relative error of the reseeding index, %; *E*′ is the relative error of the miss-seeding index, %; *n*_*0*_ is the manual hole count in one seed; *n*_*1*_ is the manual hole count for more than one seed; *n*_*2*_ is the manual hole count without seeds; *N* is the manual hole count of the total number of holes; *$n_{0}^{\prime }$* is the monitoring result for the holes in one seed; $n_{1}^{\prime }$ is the monitoring result for the holes in more than one seed; $n_{2}^{\prime }$ is the monitoring result for holes without seeds; *n*′ is the total number of sample-seeds spacings; *x* is the theoretical seed-spacing of seeding, mm; $\bar{x}$ is the average distance between sample points, mm; *S* is the relative error of the seeding numbers; *n*_*a*_ is the result of manually counting the total number of holes; and *n*_*b*_ is the monitoring result for the total number of holes.

**FIGURE 8:**
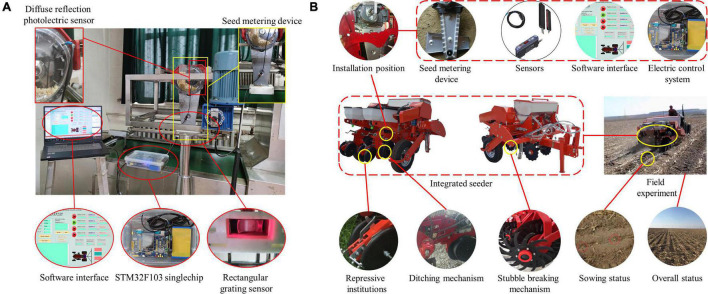
Experiment. **(A)** Bench experiment. **(B)** Field experiment.

The seed metering device was fixed on the mounting frame in the experimental process. The bed belt moved in the opposite direction relative to the seed metering device. The forward motion state of the seeder was simulated. The fuel injection pump sprayed oil on the bed belt. The corn seed fell from the seeding mouth to the seed bed belt. Real-time detection and data acquisition were carried out through the camera processing device to accurately measure the seeding performance index, as shown in Figure 8.

In May 2021, a field validation experiment was carried out in Acheng District, Harbin City, Heilongjiang Province (127.30 East longitude and 45.33 North latitude). Field environment: straw coverage is (2.08 - 3.16) kg/m^2^, straw stubble height is (130 - 150) mm, soil moisture content is 18 - 22%, and soil firmness is (16.05 - 20.41) MPa. Based on the long-belt finger-clip precision corn seed metering device and the developed monitoring system, the no tillage sowing device was integrated, as shown in Figure 8B. The power unit is John Deere 454 tractor. During the test, the rotation speed of the seed metering plate shall be consistent with the bench test, so the forward speed of the tractor shall be calibrated before the test. When the forward speed is 3, 5, 7, 9, 11, 13 km/h, the corresponding seed metering disk speed is 15, 25, 35, 45, 55, 65 r/min. The seed spacing is 220 mm. After sowing, the soil layer shall be removed manually to determine the spacing of corn seeds, and errors caused by human factors shall be avoided as far as possible. Each group of experiments was repeated 3 times, and the results were taken as the average value.

The influence of the seed metering tray rotating speed on the sensor monitoring accuracy can be comprehensively analyzed, in essence, as the influence of the sensed frequency and speed of the seed on the sensor monitoring accuracy (Maleki et al., 2006). As the seed metering tray rotated coaxially with seed guide pulley I, seed guide pulley I and the seed guide pulley rotated through the seed guide belt. The speed and frequency of the seeds passing over the two sensors were the same. To further explore the relationship between these two factors, the corresponding values were controlled by adjusting the number of finger clips and the rotation speed of the seed metering tray.

The sensed seed frequency can be obtained based on the rotating speed of the seed metering tray as follows:


(7) $$f=\frac{1}{5}\,n$$

where *f* is the seed frequency over the sensor, seeds/s; and *n* is the rotating speed of the seed metering tray, r/min.

The seed speed over the sensor was calculated (ignoring the distance between the rectangular optical fiber sensor and the bottom of the seeding guide pulley II) as follows:


(8) $$v=\frac{\pi \,R_{1}\,n}{30000}$$

where *v* is the seed speed through the sensor, m/s.

## Results

### Comparative Analysis of the Seeding Performances Between Long-Belt Finger-Clip and Conventional Finger-Clip Precision Corn Seed Metering Devices

**FIGURE 9:**
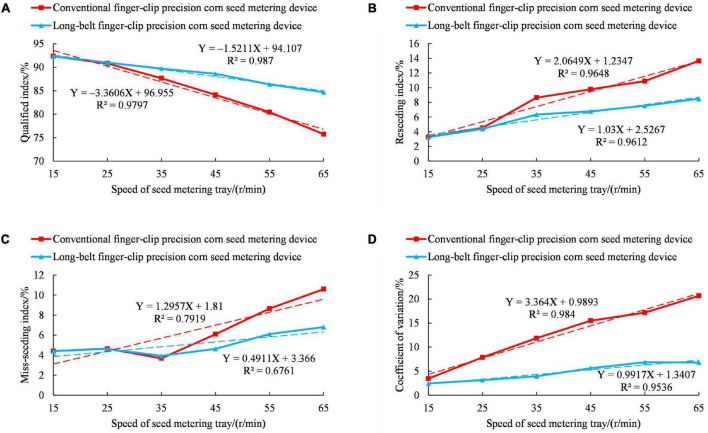
Comparative analysis of the seeding performances for the long-belt finger-clip and conventional finger-clip precision corn seed metering devices. **(A)** Influence of the rotating speed of the seed metering tray on the qualified index. **(B)** Influence of the rotating speed of the seed metering tray on the reseeding index. **(C)** Influence of the rotating speed of the seed metering tray on the miss-seeding index. **(D)** Influence of the rotating speed of the seed metering tray on the coefficient of variation.

The comparison of the seeding performance between the long-belt finger-clip precision corn metering device and the conventional finger-clip precision corn seed metering device is shown in Figure 9. The regression equation in Figure 9 is used to analyze and predict the relationship between performance and seed metering tray speed. The greater the slope of regression equation, the greater the change of seed metering performance index with factors. R^2^ represents the overall fit of the regression equation. The maximum value of R^2^ is 1. The greater R^2^, the better the fitting degree of the regression equation. Figure 9 shows that the seeding performances of the long-belt finger-clip precision corn seed metering device and the conventional finger-clip precision corn seed metering device decreased with increasing speed for the seed metering tray. When the rotational speed of the seed metering tray was 15-25 r/min, the slope of the coefficient of variation for the conventional finger-clip precision corn seed metering device is greater than that of the long-belt finger-clip precision corn seed metering device. The results showed that with the increase in the rotational speed of the seed plate, the stability of the conventional finger clip precision corn seed metering device and the consistency between the seed spacing were poor. When the rotational speed of the seed metering tray was 25-65 r/min, the qualified index of the conventional finger-clip precision metering device decreased greatly, from 90.83 to 75.75%. The reseeding index, the miss-seeding index and coefficient of variation increased greatly. The reseeding index increased from 4.52 to 13.66%, the miss-seeding index increased from 4.65 to 10.59%, and the coefficient of variation increased from 7.88 to 20.69%. However, the qualified index, the reseeding index, the miss-seeding index and coefficient of variation in the long-belt finger-clip precision corn seed metering device changed, and the overall change was relatively stable. When the rotation speed of seed metering disk is 65 r/min, the qualification index, reseeding index, miss-seeding index and variation coefficient are 84.70, 8.49, 6.81, and 6.83%, respectively. The results showed that the long-belt finger-clip precision seed metering device can smoothly transport and deliver corn seeds and effectively reduce the reseeding index, miss-seeding index and coefficient of variation compared to the conventional finger-clip precision seed metering device.

### Influence of the Rotation Speed of the Seed Metering Tray on the Monitoring Accuracy of the Sensor

**FIGURE 10:**
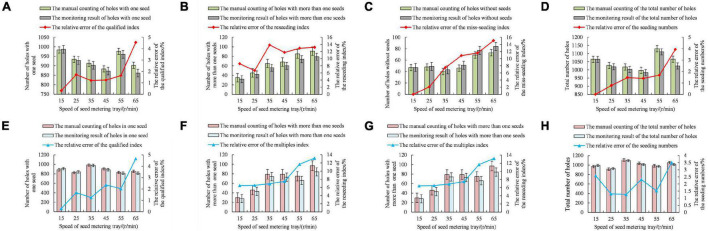
Influence of the rotating speed of the seed metering tray on the monitoring accuracy of the sensor. **(A)** Qualified number in bench experiment. **(B)** Reseeding number in bench experiment. **(C)** Miss-seeding number in bench experiment. **(D)** Seeding amount in bench experiment. **(E)** Qualified number in field experiment. **(F)** Reseeding number in field experiment. **(G)** Miss-seeding number in field experiment. **(H)** Seeding amount in field experiment.

To explore the influence of the rotating speed of the seed metering tray on the monitoring accuracy of the sensor, the qualified number, the multiple seeding number, the miss seeding number and the seeding number monitored by the sensor were compared to the data obtained by manual measurement. The variation rules for the relative errors of the qualified index, the reseeding index, the miss-seeding index and seeding amount with the rotating speed of the seed metering tray are shown in Figure 10.

Figure 10A shows that the relative errors of the qualification index, the reseeding index, the miss-seeding index and seeding amount increased gradually with increasing speed for the seed metering tray in the bench experiment. The results showed that the monitoring accuracy of the sensor decreased with the increasing rotation speed of the seed metering tray. The relative error of the reseeding index and the miss-seeding index was larger than that of the qualified index and seeding amount, mainly due to the small number of multiple seeding holes, and the miss seeding holes were larger as a whole when compared with the manually measured data. However, the overall trend reflected the changing law of the sensor monitoring accuracy.

Figure 10B shows that the relative errors of the qualification index, the reseeding index, the miss-seeding index increased gradually with increasing speed for the seed metering tray in the field experiment. It is verified that the monitoring accuracy of the sensor decreases gradually with the increase of the rotation speed of the seed metering disk. Table 2 is the comparison results between bench experiment and field experiment of long-belt finger-clip precision corn seed metering device and monitoring system. The performance of the seed metering device in the field experiment is slightly lower than that in the bench experiment, but the data obtained by the monitoring system is less different from that in the bench experiment, and the performance is relatively stable.

**TABLE 2:** Comparison results between bench experiment and field experiment of long-belt finger-clip precision corn seed metering device and monitoring system.

Speed of seed metering tray (r/min)		15	25	35	45	55	65
Performance of long-belt finger-clip precision corn seed metering device	Qualified index in bench experiment (%)	92.34	90.99	89.72	88.6	86.35	84.7
	Qualified index in field experiment (%)	91.46	90.38	89.24	87.65	84.33	81.26
	Reseeding index in bench experiment (%)	3.26	4.38	6.35	6.77	7.54	8.49
	Reseeding index in field experiment (%)	3.11	5.02	7.12	7.65	7.61	9.23
	Miss-seeding index in bench experiment (%)	4.40	4.63	3.93	4.63	6.11	6.81
	Miss-seeding index in field experiment (%)	5.43	4.60	3.64	4.70	8.06	9.51
	Coefficient of variation in bench experiment (%)	2.46	3.15	3.93	5.63	6.87	6.83
	Coefficient of variation in field experiment (%)	2.39	3.27	4.02	5.54	6.96	7.8
Performance of monitoring system	The relative error of qualified index in bench experiment (%)	0.31	1.71	1.2	1.24	1.63	4.55
	The relative error of qualified index in field experiment (%)	0.28	1.68	1.25	2.36	2.03	4.67
	The relative error of reseeding index in bench experiment (%)	8.57	6.67	13.85	11.76	12.94	13.19
	The relative error of reseeding index in field experiment (%)	6.45	6.52	6.89	7.5	11.67	13.15
	The relative error of miss-seeding index in bench experiment (%)	0	2.08	7.5	10.87	11.59	15.07
	The relative error of miss-seeding index in field experiment (%)	3.13	3.03	7.31	7.14	13.46	15.71
	The relative error of seeding numbers in bench experiment (%)	0	0.78	1.47	1.4	1.68	3.94
	The relative error of seeding numbers in field experiment (%)	2.58	1.31	1.26	2.32	1.52	3.52

The seeding frequency, seeding speed and speed of the seed metering tray are shown in Table 3.

**TABLE 3:** Seeding frequency and speed corresponding to the rotating speed of the seed metering tray.

Level	Speed of seed metering tray (r/min)	Seeding frequency (seeds/s)	Seeding speed (m/s)
1	15	3	0.09
2	25	5	0.15
3	35	7	0.22
4	45	9	0.28
5	55	11	0.35
6	65	13	0.41

To further explore the influences of the seeding frequency and seeding speed on the monitoring accuracy of the sensor, the relative errors of the qualified index, the reseeding index, the miss-seeding index and seeding amount were used as evaluation parameters. Through the value of fixed level 4 (Wang et al., 2017), single factor experiments were carried out on the seeding frequency and seeding speed.

**FIGURE 11:**
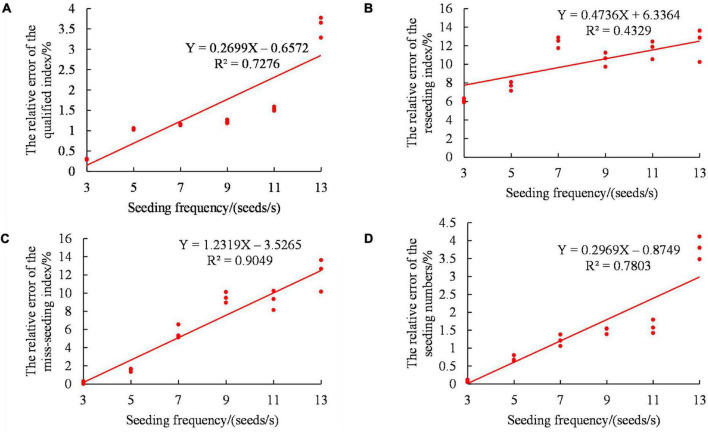
Influence of the seeding frequency on the monitoring accuracy of the sensor. **(A)** Relative error of the qualified index. **(B)** Relative error of the reseeding index. **(C)** Relative error of the miss-seeding index. **(D)** Relative error of the seeding amount.

First, linear regression analysis of the metering frequency was carried out. Figure 11 shows the linear regression analysis of the seeding frequency to the evaluation parameters. As shown in Figure 11, the relative errors of the qualified index, multiple seeding, miss-seeding index and seeding amount all increased gradually with increasing seeding frequency. At the same time, the relative measurement error was relatively scattered, and the overlap points decreased with increasing seeding frequency. The monitoring stability of the sensor decreased gradually. This may have occurred because under the condition of a fixed seeding speed, the greater the seeding frequency was, the smaller the time interval between the two seeds was. This, in turn, led to the increase in the seed state and the connection between the seeds in the state of the multiple seeding. The probability of multiple seeding and missed seeding increased, and the probability of sensor misjudgment increased.

To clarify the significance of the sensor monitoring accuracy under different seeding frequencies, Design-Expert 8.0.6 software was used to analyze the frequencies of the seeds passing over the sensor, as shown in Table 4. The P values of the qualified index relative error, the multiple seeding relative error, the miss-seeding index relative error and the seeding amount relative error were all less than 0.0001. The results showed that the influence of the seeding frequency on the evaluation parameters was very significant.

**TABLE 4:** Analysis of seeding frequency variance based on the evaluation parameters.

Evaluation parameters	Source of variation	Sum of squares	df	Mean square	*F*-value	*P*-value
The relative error of the qualified index	Seeding frequency	18.41	5	3.68	312.15	< 0.0001
	Pure Error	0.14	12	0.012		
	Total variation	18.55	17			
The relative error of the reseeding index	Seeding frequency	103.21	5	20.64	23.36	< 0.0001
	Pure Error	10.60	12	0.88		
	Total variation	113.81	17			
The relative error of the miss-seeding index	Seeding frequency	342.12	5	68.42	76.94	< 0.0001
	Pure Error	10.67	12	0.89		
	Total variation	352.79	17			
The relative error of the seeding numbers	Seeding frequency	23.94	5	4.79	163.43	< 0.0001
	Pure Error	0.35	12	0.029		
	Total variation	24.29	17			

**FIGURE 12:**
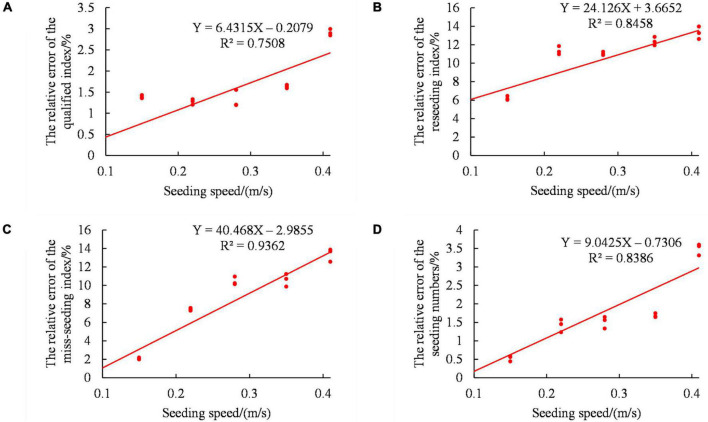
Influence of the seeding speed on the monitoring accuracy of the sensor. **(A)** Relative error of the qualified index. **(B)** Relative error of the reseeding index. **(C)** Relative error of the miss-seeding index. **(D)** Relative error of the seeding amount.

Linear regression analysis of the seeding speed was carried out. Figure 12 shows the linear regression analysis of the seeding speed to the evaluation parameters. As shown in Figure 12, the relative errors of the qualified index, multiple seeding, miss-seeding index and seeding amount all increased gradually with increasing seeding speed. At the same time, the relative error of measurement was relatively concentrated, and there were more overlap points with increasing seeding speed. Although the monitoring accuracy of the sensor had a downward trend, the stability did not fluctuate greatly with increasing seeding speed. This may be because under the condition of fixed seeding frequency, the greater the seed metering speed was, the faster the opening and closing speed of the seed-pick finger-clip was. This, in turn, led to a decrease in the stability of the seed from the seed-pick finger-clip to the seed guide belt. The counting accuracy of the diffuse reflection photoelectric sensor and rectangular optical fiber sensor was reduced. This was closely related to the structure and working principle of the finger-clip corn seed metering device.

To clarify the significance of the sensor monitoring accuracies under different seeding speeds, Design-Expert 8.0.6 software was used to analyze the single factor variance in the speed of seeds passing over the sensor, as shown in Table 5. The P values of the qualified index relative error, the multiple seeding relative error, the miss-seeding index relative error and the seeding amount relative error were all less than 0.0001. The results showed that the influence of the seeding speed on the evaluation parameters was very significant.

**TABLE 5:** Analysis of the seeding speed variance based on the evaluation parameters.

Evaluation parameters	Source of variation	Sum of squares	df	Mean square	*F*-value	*P*-value
The relative error of the qualified index	Seeding speed	12.15	5	2.43	256.43	< 0.0001
	Pure Error	0.11	12	0.009472		
	Total variation	12.26	17			
The relative error of the reseeding index	Seeding speed	170.83	5	34.17	203.29	< 0.0001
	Pure Error	2.02	12	0.17		
	Total variation	172.85	17			
The relative error of the miss-seeding index	Seeding speed	414.15	5	82.83	407.54	< 0.0001
	Pure Error	2.44	12	0.20		
	Total variation	416.59	17			
The relative error of the seeding numbers	Seeding speed	20.79	5	4.16	262.90	< 0.0001
	Pure Error	0.19	12	0.016		
	Total variation	20.98	17			

## Discussion

As the most widely used mechanical seed metering device, the improvement and effective monitoring of the sowing quality of finger-clip precision corn seed metering device is of great value to promote the development of the sowing link of precision agriculture. The conventional finger-clip precision corn seed metering device is equipped with a seed guide tube under it, so it is unable to sowing at zero speed, resulting in the reduction of seed metering performance when the seeds bounce and collide. In addition, the structural configuration cannot meet the layout requirements of sensors. In this study, the seed guide tube structure was replaced by the lengthened seed guide belt, which simplified the overall structure. At the same time, the research was carried out from the aspect of stable delivery mechanism to achieve the effect of zero speed seed delivery. Due to the limitation of the structure and metering mode of finger-clip precision corn seed metering device, the traditional single sensor monitoring cannot meet its monitoring requirements. Based on multi-sensor information fusion, a diffuse reflection photoelectric sensor and a rectangular grating sensor were configured at the seed guide and seed feeding positions, respectively, which solved the problem that the traditional single seed metering device cannot judge the seed overlap, and effectively improved the monitoring accuracy. In addition, based on C#, a software interactive interface which was easy for secondary development was designed. Comprehensive bench test and field test verified the effectiveness and accuracy of seed metering quality and monitoring system. This study provides ideas for the optimal design scheme of zero speed seeding at a low position, and provides reference for the development of multi-sensor fusion monitoring system in sowing link.

The performance of mechanical seed metering device is determined by its seed metering mode, seed guiding characteristics and inherent properties. Kocher et al. (2011) pointed out that the uniformity index of seeds is determined by the seed guide tube. The convention finger-clip precision corn seed metering device is equipped with a seed guide tube under it, so it is unable to seed at zero speed, resulting in the reduction of seed metering performance when the seeds bounce and collide. When the rotation speed of the conventional finger-clip precision corn seed metering device was 45r/min, the coefficient of variation was 17.97% (Wang et al., 2017). In this study, the bench test of conventional finger-clip precision corn seed metering device was carried out, and the research results were basically the same, which was 15.51%. The seed guide tube structure was replaced by the lengthened seed guide belt, which simplified the overall structure. At the same time, the research was carried out from the aspect of stable delivery mechanism to achieve the effect of zero speed seed delivery. The coefficient of variation decreased to 5.63% when the speed of seed metering disk was 45 r/min. In addition, the rotation speed of the seed metering disk was increased. When the rotation speed of the seed metering disk was 65 r/min, the qualificated index was 84.7%, the reseeding index was 8.49%, the miss-seeding index was 6.81%, and the coefficient of variation was 6.83%. If the seed metering performance needs to be further improved, the seed metering mode needs to be changed or the reseeding device needs to be added.

If a single sensor is used for monitoring, the partially overlapped adjacent seeds cannot generate photoelectric signal interval pulse, and the system cannot monitor the seed falling time difference, resulting in missed judgment of overlapped seeds. When the rotation speed of seed metering disk was 36 r/min, the monitoring accuracy of missed sowing was 85.6% (Ji et al., 2016). In this study, a seed metering quality monitoring system is developed based on multi-sensor fusion. The high seed metering speed leads to the decline of sensor monitoring accuracy, which is consistent with the change trend of using infrared sensor developed by Karimi et al. (2019). When the rotation speed of seed metering disk was 35r/min, the monitoring accuracy of missed sowing was 92.50%, which effectively improved the monitoring accuracy of missed sowing. In addition, in order to facilitate debugging, the monitoring system collects data through the USB to TTL chip on the upper computer. With the promotion and popularization of precision agriculture, more and more on-board computers will be configured on tractors. Later, this study will be transplanted to on-board computers for practical operation.

The speed of the planter has an important influence on the sowing performance. With the increase of speed, the grain spacing increases, the qualified index decreases and the miss-seeding index increases (Cay et al., 2018; Yang et al., 2019). The speed of planter directly determines the speed of seed tray, which is consistent with the results of this study. The speed of seed tray not only has an impact on seed metering performance, but also has a certain impact on seeds. The higher the speed of the seed tray, the greater the impact on the seed when clamping the seed, which is easy to cause seed damage. Staggenborg et al. (2004) pointed out that the increase of operation speed will also reduce the corn yield. In addition, bench experiment and field experiment was conducted, and the comparison results was shown in Table 2. The field experiment has little influence on the accuracy of the monitoring system and has a great influence on the performance of the seed metering device. Under the same rotating speed, the seed metering performance of field experiment was lower than that of bench experiment. This is mainly due to the uneven field ground, complex and uncontrollable vibration, which leads to the change of seed posture in the clamping process, resulting in the phenomenon of reseeding and miss-seeding. In the later stage, we will focus on the in-depth research from the corn seed posture as the starting point to explore the seed damage mechanism under different seed metering disk speed and the seed falling posture under different vibration frequency. In addition, the growth state of maize from sowing to harvest will be tracked to explore the corn yield under different working conditions.

## Conclusion

Through the optimization of the conventional finger-clip precision corn seed metering device, a long-belt structure was incorporated in this study. The overall structure and working principle of the long-belt finger-clip precision corn seed metering device were described, the mechanism of stable transportation and delivery was analyzed, and a corn seeding quality monitoring system was designed. The conclusions were as follows:

(1). The seeding performance of the long-belt finger-clip precision corn seed metering device and the conventional finger-clip precision corn seed metering device decreased with increasing speed for the seed metering tray. Compared with the conventional finger clip precision corn metering device, when the rotation speed of the seed metering disk was 65r/min, the qualified index of the long-belt finger-clip precision corn seed metering device increased from 75.75 to 84.70%, the reseeding index decreased from 13.66 to 8.49%, the miss-seeding index decreased from 10.59 to 6.81%, and the coefficient of variation decreased from 20.69 to 6.83%. The long-belt finger-clip precision corn seed metering device can transport and deliver corn seeds smoothly and effectively to reduce the reseeding index, the miss-seeding index and the coefficient of variation.

(2). The relative error of the qualified index, the reseeding index, the miss-seeding index increased with increasing seed metering tray speed. In the field experiment, the relative deviation of qualified index increased from 0.28 to 4.67%, the relative deviation of reseeding index increased from 6.45 to 13.15%, and the relative deviation of miss-seeding index increased from 3.13 to 15.71%. The monitoring accuracy of the sensor decreased gradually.

(3). The effects of the seeding frequency and seeding speed on the four evaluation parameters were very significant. With increasing seeding frequency, the relative errors of the qualified index, the reseeding index, the miss-seeding index and seeding amount all increased gradually, and the monitoring stability of the sensor decreased gradually. With increasing seeding speed, the relative errors of the qualified index, the reseeding index, the miss-seeding index and seeding amount all increased gradually, but the monitoring stability of the sensor was basically unchanged.

## Data Availability Statement

The original contributions presented in the study are included in the article/supplementary material, further inquiries can be directed to the corresponding author/s.

## Author Contributions

HT, CX, ZW, and JW designed and performed the experiments and analyzed the data. HT, CX, and QW wrote the manuscript. All authors contributed to the article and approved the submitted version.

## Conflict of Interest

The authors declare that the research was conducted in the absence of any commercial or financial relationships that could be construed as a potential conflict of interest.

## Publisher’s Note

All claims expressed in this article are solely those of the authors and do not necessarily represent those of their affiliated organizations, or those of the publisher, the editors and the reviewers. Any product that may be evaluated in this article, or claim that may be made by its manufacturer, is not guaranteed or endorsed by the publisher.
